# Supplementary material for: Dinosaur diversification rates were not in decline prior to the K-Pg boundary
Source: R Soc Open Sci. 2020 Nov 18;7(11):201195. doi: 10.1098/rsos.201195 (PMC7735361; doi:10.1098/rsos.201195)
Supplement: Supplementary methods [file rsos201195supp1.docx]

# Supplementary Materials for:

# *Dinosaur diversification rates were not in decline prior to the K-Pg boundary.*

# *Joseph A. Bonsor, Paul M. Barrett, Thomas J. Raven and Natalie Cooper*

# SUPPLEMENTAL METHODS

# Phylogenetic tree parameters

None of the nine trees had available tree files so we used their character matrices to re-run their phylogenetic analyses and obtain tree topologies. We used TNT v. 1.5 (Goloboff *et al*. 2008) with the search settings of the original analysis and produced consensus trees of each. Details of the settings for each tree are below. Note that our aim was to replicate the phylogenies produced by the taxon-expert authors of the papers below, rather than aiming to infer the phylogenies *de novo*. Therefore we follow these authors’ protocols, including how they performed the tree searches, which taxa they removed, and what kind of consensus tree they used.

Arbour *et al*. 2016

No characters ordered or weighted.

Excluded *Tianchisaurus, Bissektipelta, Minmi paravertebra, Anoplosaurus, Acanthopholis, Dracopelta,* and *Horshamosaurus*.

Heuristic searches on Wagner trees using TBR branch swapping with 1000 random addition sequences holding 10ntrees per replicate continuing subsequent TBR swapping on all stored minimum length trees and with a maximum set of trees of 10000.

50% majority-rule consensus tree.

No taxa removed before dating.

Chiba *et al*. 2018

No details given in original manuscript or SI.

*Leptoceratops gracilis* outgroup

Heuristic search with 1000 replicates of Wagner trees using random addition sequences, TBR branch swapping, 1000 trees saved per replicate.

Strict consensus tree.

No taxa removed before dating.

Carbadillo *et al*. 2017

From the multistate characters, 24 were treated as ordered (14, 61, 100, 102, 109, 115, 127, 132, 135, 136, 166, 179, 195, 256, 259, 276, 277, 278, 279, 299, 303, 346, 352, and 354).

No characters weighted.

*Plateosaurus engelhardti* outgroup.

Heuristic tree search with 10000 replicates of Wagner trees (with random addition sequence of taxa).

TBR branch swapping, 1000 trees saved per replicate.

Strict consensus with *Lusotitan, Padillasaurus, Ligabuesaurus, Wintonotitan, Nemegtosaurus* and *Trigonosaurus* removed.

Cau *et al*. 2015

No characters ordered or weighted.

*Herrerasaurus* outgroup.

New Technology search with 100 replicates

Parameters set using default values.

TBR heuristic searches with 1000 replicates.

No more than 99999 trees were saved.

Strict consensus tree.

Modern birds: *Crypturellus undulatus, Gallus gallus, Crax pauxi, Anas platyrhynchos* and *Chauna torquata* removed prior to dating.

Cruzado-Caballero & Powell 2017

No characters ordered or weighted.

*Ouranosaurus nigeriensis* outgroup.

Heuristic search with 1000 replicates of Wagner trees using random addition sequences, TBR branch swapping, 1000 trees saved per replicate.

Strict consensus tree.

No taxa removed before dating.

Gonzalez Riga *et al*. 2018

Characters 11, 14, 15, 27, 40, 51, 104, 122, 147, 148, 177, 195, 205, 259 ordered multistate.

Characters equally weighted.

Excluded *Astrophocaudia, Australodocus, Brontomerus, Fukuititan, Fusuisaurus, Liubangosaurus, Monoglosaurus, and Tendaguria.*

Analysed using stabilise consensus option in New Tech search using sectorial, drift and tree fusing, with consensus stabilised five times, prior to using resultant trees as starting trees for traditional search using TBR.

Strict consensus tree.

No taxa removed before dating.

Mallon *et al*. 2016

No characters ordered or weighted.

No taxa excluded *a priori*.

*Leptoceratops* outgroup.

Branch and bound search algorithm.

Originally used either traditional homology or new homology scheme – same dataset but different scores, for this used traditional homology as produced better resolution in results. Strict reduced consensus tree with *Bravoceratops* and *Eotriceratops* removed.

No taxa removed before dating.

Raven & Maidment 2017

Characters 1–24 continuous, 105 and 106 ordered.

No characters weighted.

No taxa excluded *a priori*.

*Pisanosaurus* outgroup.

New tech search with sectorial, ratchet, drift and tree fusing. 10 random addition sequences. Then TBR search with 1 random addition seed and 1000 replicates.

One MPT produced so no need for a consensus tree.

No taxa removed before dating.

Thompson *et al*. 2012

Characters 25, 27, 32, 133, 159 and 167 removed.

No characters ordered or weighted.

Excluded *Bissektipelta.*

*Lesothosaurus* outgroup.

Traditional search with 1000 replicates, TBR and 100 trees saved per replicate.

Strict consensus tree.

Removed *Minotaurasaurus ramachandrani* before dating as its is provenance unknown so no date is available.

### **Dating the trees**

We dated all trees using the timePaleoPhy function from the R (R Core Team 2019) package paleotree version 3.1.0 (Bapst 2012), incorporating uncertainty by drawing taxon ages randomly from a uniform distribution between their maximum and minimum possible ages (timePaleoPhy option: dateTreatment = ‘minMax’). We followed this dating procedure to match how the trees used in Sakamoto *et al.* (2016) were dated. Zero length branches were lengthened by imposing a minimum branch duration of 1 Ma (timePaleoPhy options: vartime = 1, type = ‘mbl’; Laurin 2004). Because this procedure randomly samples ages we exported 100 dated trees and performed analyses across all the trees.

It is possible that dating the trees in this way may result in artificially old root ages. To test for this, we extracted the root age for each of the 900 trees dated in this study using paleotree (Bapst *et al.* 2012). The distribution of root ages across each of the 100 trees for each phylogeny are shown in S6. The dotted black line shows the oldest inferred origin date for the clade, i.e. 201.3 Ma (Early Jurassic) for Ornithischia and 231.4 Ma (Carnian) for Sauropodomorpha and Theropoda. The red and blue dotted lines show the oldest and youngest ages respectively of taxa in the trees.

All of the trees have younger (rather than older) root ages, with the exception of Raven & Maidment’s (2017) ornithischian tree which contained outgroup taxa that are older than the group, and the dates instead fall within the oldest and youngest ages of the taxa in the tree. This suggests that our dating method is not biasing trees to be too old and thus the minimum branch length of 1 million years is appropriate for the analyses we present here.

# SUPPLEMENTARY TABLES

*Tables S1–S3: Full results from all models for the 900 trees from this study, plus the nine trees from Sakamoto et al. (2016) are available in associated csv files, one for models estimating intercepts (S1), one where intercepts were set to zero (S2), and one where intercepts were set to 1.0 (S3).*

***The column names in Tables S1-S3 correspond to the following.***

- **tree.ID** = ID number of tree (1-900).
- **tree** = name of tree.
- **null_DIC** = Deviance information criterion for the null model (Figure 1A).
- **slow_DIC** = Deviance information criterion for the downturn model (Figure 1C).
- **asym_DIC** = Deviance information criterion for the slowdown to asymptote model (Figure 1B).
- **null_post_mean_intercept** = Mean intercept from the posterior for the null model.
- **null_lower95_CI_intercept** and **null_upper95_CI_intercept** = 95% confidence intervals on the mean intercept for the null model.
- **null_post_mean** = Mean value of the time elapsed parameter from the posterior for the null model.
- **null_lower95_CI** and **null_upper95_CI** = 95% confidence intervals on the mean time elapsed parameter for the null model.
- **null_ess** = Effective sample size for the null model.
- **null_pMCMC** = p value for the null model.
- **slow_post_mean_intercept** = Mean intercept from the posterior for the downturn model.
- **slow_lower95_CI_intercept** and **slow_upper95_CI_intercept** = 95% confidence intervals on the mean intercept for the downturn model.
- **slow_post_mean** = Mean value of the time elapsed parameter from the posterior for the downturn model.
- **slow_lower95_CI** and **slow_upper95_CI** = 95% confidence intervals on the mean time elapsed parameter for the downturn model.
- **slow_post_mean_2** = Mean value of the time elapsed^2^ parameter from the posterior for the downturn model.
- **slow_lower95_CI_2** and **slow_upper95_CI_2** = 95% confidence intervals on the mean time elapsed^2^ parameter for the downturn model.
- **slow_ess** = Effective sample size for the downturn model.
- **slow_pMCMC** = p value for the downturn model.
- **asym_post_mean_intercept** = Mean intercept from the posterior for the slowdown to asymptote model.
- **asym_lower95_CI_intercept** and **asym_upper95_CI_intercept** = 95% confidence intervals on the mean intercept for the slowdown to asymptote model.
- **asym_post_mean_sqrt** = Mean value of the √time elapsed parameter from the posterior for the slowdown to asymptote model.
- **asym_lower95_CI_sqrt** and **asym_upper95_CI_sqrt** = 95% confidence intervals on the mean √time elapsed parameter for the slowdown to asymptote model.
- **asym_ess** = Effective sample size for the slowdown to asymptote model.
- **asym_pMCMC** = p value for the slowdown to asymptote model.

*Table S4: A comparison of the best model results from Sakamoto et al. (2016; Table S01) and this study for the two Benson et al. (2014) trees and Lloyd et al. (2008) tree. Note that the analyses are Bayesian so we do not expect them to be identical. FAD = first occurrence date; LAD = last occurrence date; DIC = deviance information criterion.*

| **tree** | **dates** | **study** | **null DIC** | **asymptoteDIC** | **downturnDIC** | **best model (ΔDIC > 4)** |
| --- | --- | --- | --- | --- | --- | --- |
| Benson1 | FAD | Sakamoto | 3507 | 3499 | 3496 | asymptote/downturn |
|  |  | this study | 3478 | 3468 | 3467 | asymptote/downturn |
|  | LAD | Sakamoto | 3463 | 3454 | 3450 | asymptote/downturn |
|  |  | this study | 3489 | 3482 | 3479 | asymptote/downturn |
|  | midpoint | Sakamoto | 3477 | 3468 | 3466 | asymptote/downturn |
|  |  | this study | 3521 | 3512 | 3509 | asymptote/downturn |
| Benson2 | FAD | Sakamoto | 3459 | 3449 | 3446 | asymptote/downturn |
|  |  | this study | 3442 | 3430 | 3429 | asymptote/downturn |
|  | LAD | Sakamoto | 3425 | 3414 | 3412 | asymptote/downturn |
|  |  | this study | 3450 | 3440 | 3438 | asymptote/downturn |
|  | midpoint | Sakamoto | 3435 | 3425 | 3421 | asymptote/downturn |
|  |  | this study | 3472 | 3463 | 3461 | asymptote/downturn |
| Lloyd | FAD | Sakamoto | 2395 | 2385 | 2382 | asymptote/downturn |
|  |  | this study | 2408 | 2398 | 2397 | asymptote/downturn |
|  | LAD | Sakamoto | 2400 | 2390 | 2388 | asymptote/downturn |
|  |  | this study | 2397 | 2388 | 2387 | asymptote/downturn |
|  | midpoint | Sakamoto | 2389 | 2379 | 2377 | asymptote/downturn |
|  |  | this study | 2402 | 2394 | 2392 | asymptote/downturn |

# SUPPLEMENTARY FIGURES


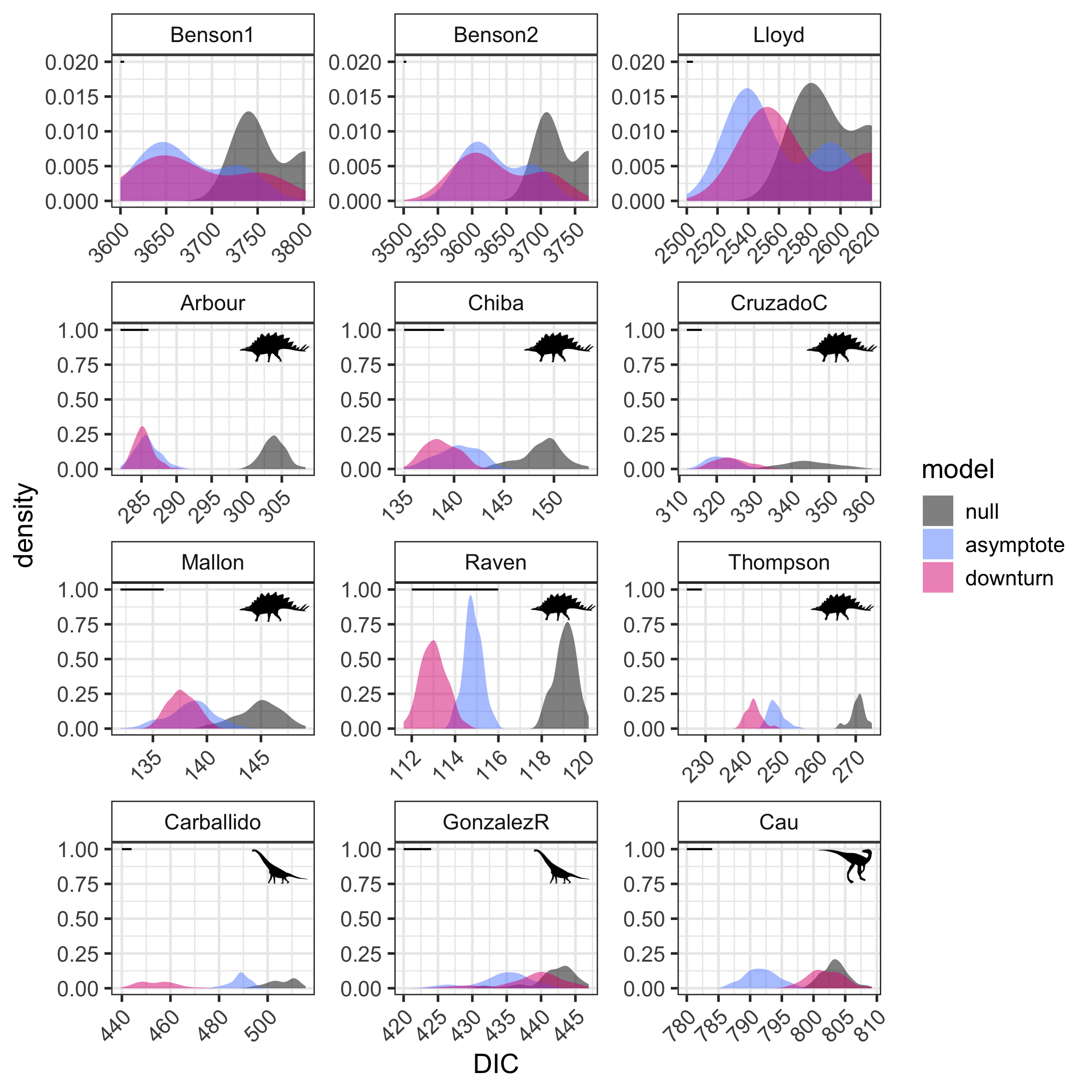


*Figure S1: DIC for the three models (see Figure 1) for each of 900 trees in this study, plus nine trees from Sakamoto et al. (2016), with intercepts set to zero. Horizontal lines show the length of 4 DIC units (note that the x-axis differs on each plot), the difference required for one model to be preferred over another. The y-axis is smaller in the first three panels as these show results from models fitted to three trees only whereas the later panels used 100 trees (see text). Panels are ordered based on the dinosaur group in each tree as follows. Dinosauria: Benson et al. (2014: two trees) and Lloyd et al. (2008); Ornithischia: Arbour et al. (2016), Chiba et al. (2018), Cruzado-Caballero et al. (2017), Mallon et al. (2016); Raven & Maidment (2017), Thompson et al. (2012); Sauropodomorpha: Carballido et al. (2017), Gonzàlez Riga et al. (2018); Theropoda: Cau et al. (2015).*


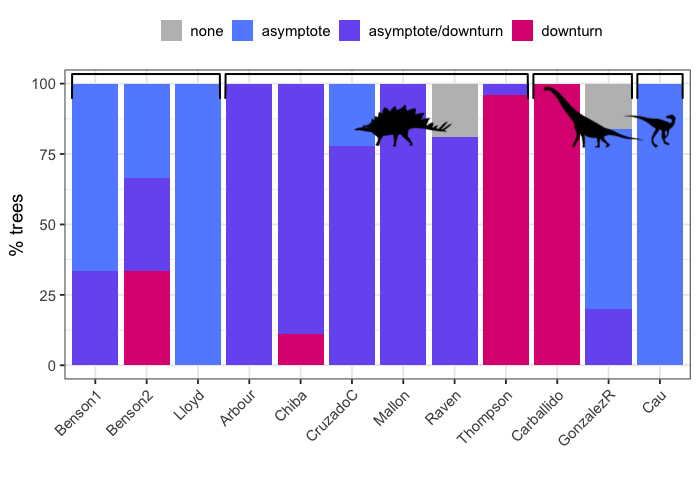


*Figure S2: The “best” model (see Figure 1) based on ΔDIC >4 units for each of 900 trees in this study, plus nine trees from Sakamoto et al. (2016), with intercepts set to zero. Bars are grouped based on the dinosaur group in each tree as follows. Dinosauria: Benson et al. (2014: two trees) and Lloyd et al. (2008); Ornithischia: Arbour et al. (2016), Chiba et al. (2018), Cruzado-Caballero et al. (2017), Mallon et al. (2016); Raven & Maidment (2017), Thompson et al. (2012); Sauropodomorpha: Carballido et al. (2017), Gonzàlez Riga et al. (2018); Theropoda: Cau et al. (2015).
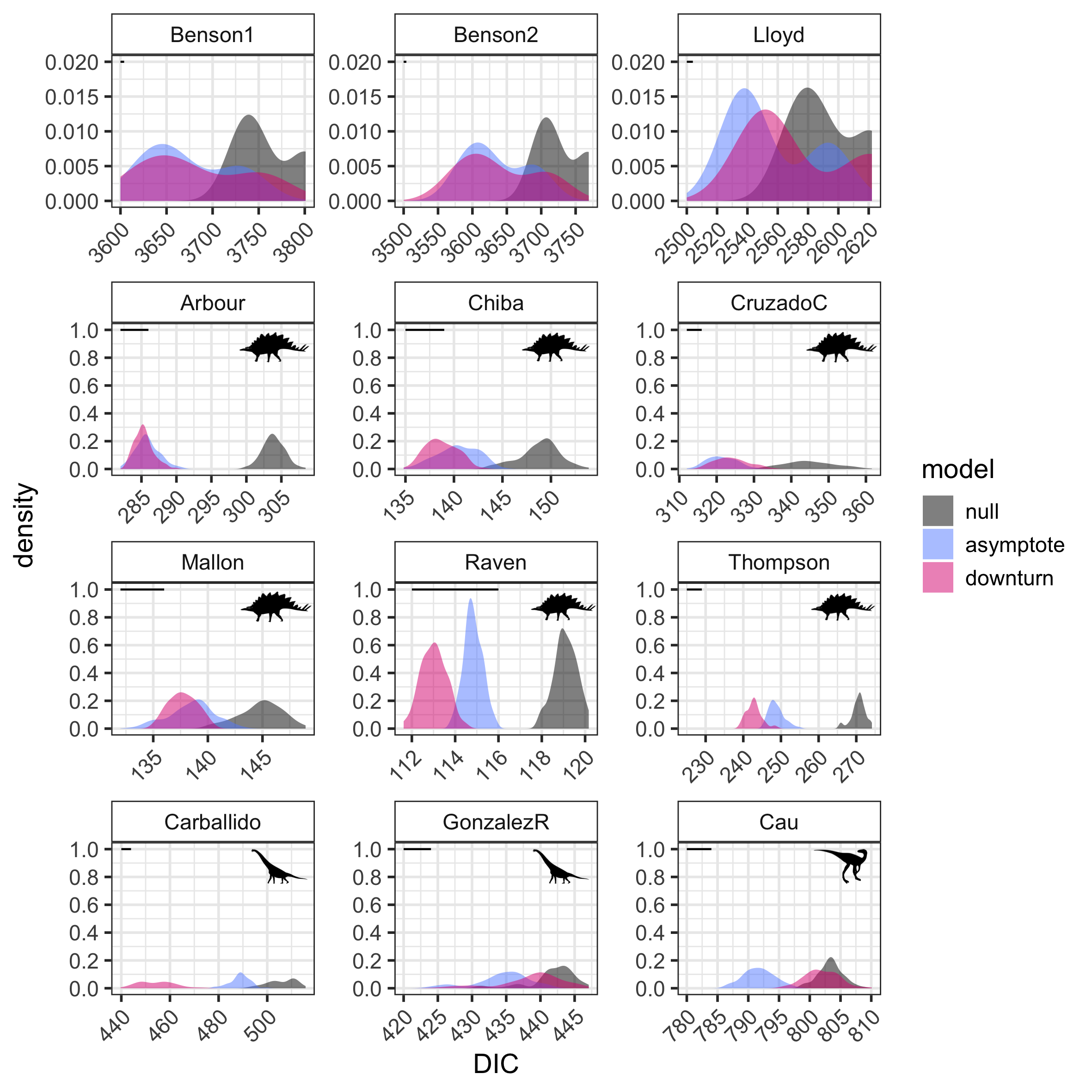
*

*Figure S3: DIC for the three models (see Figure 1) for each of 900 trees in this study, plus nine trees from Sakamoto et al. (2016), with intercepts set to 1.0. Horizontal lines show the length of 4 DIC units (note that the x-axis differs on each plot), the difference required for one model to be preferred over another. The y-axis is smaller in the first three panels as these show results from models fitted to three trees only whereas the later panels used 100 trees (see text). Panels are ordered based on the dinosaur group in each tree as follows. Dinosauria: Benson et al. (2014: two trees) and Lloyd et al. (2008); Ornithischia: Arbour et al. (2016), Chiba et al. (2018), Cruzado-Caballero et al. (2017), Mallon et al. (2016); Raven & Maidment (2017), Thompson et al. (2012); Sauropodomorpha: Carballido et al. (2017), Gonzàlez Riga et al. (2018); Theropoda: Cau et al. (2015).*

*
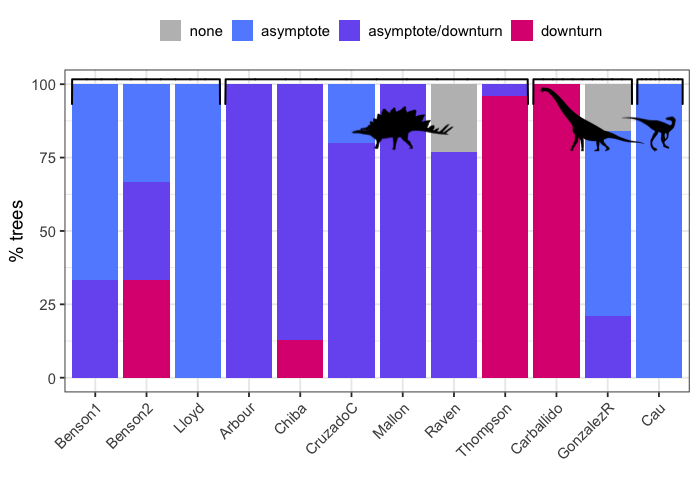
Figure S4: The “best” model (see Figure 1) based on ΔDIC >4 units for each of 900 trees in this study, plus nine trees from Sakamoto et al. (2016), with intercepts set to 1.0. Bars are grouped based on the dinosaur group in each tree as follows. Dinosauria: Benson et al. (2014: two trees) and Lloyd et al. (2008); Ornithischia: Arbour et al. (2016), Chiba et al. (2018), Cruzado-Caballero et al. (2017), Mallon et al. (2016); Raven & Maidment (2017), Thompson et al. (2012); Sauropodomorpha: Carballido et al. (2017), Gonzàlez Riga et al. (2018); Theropoda: Cau et al. (2015).*

*
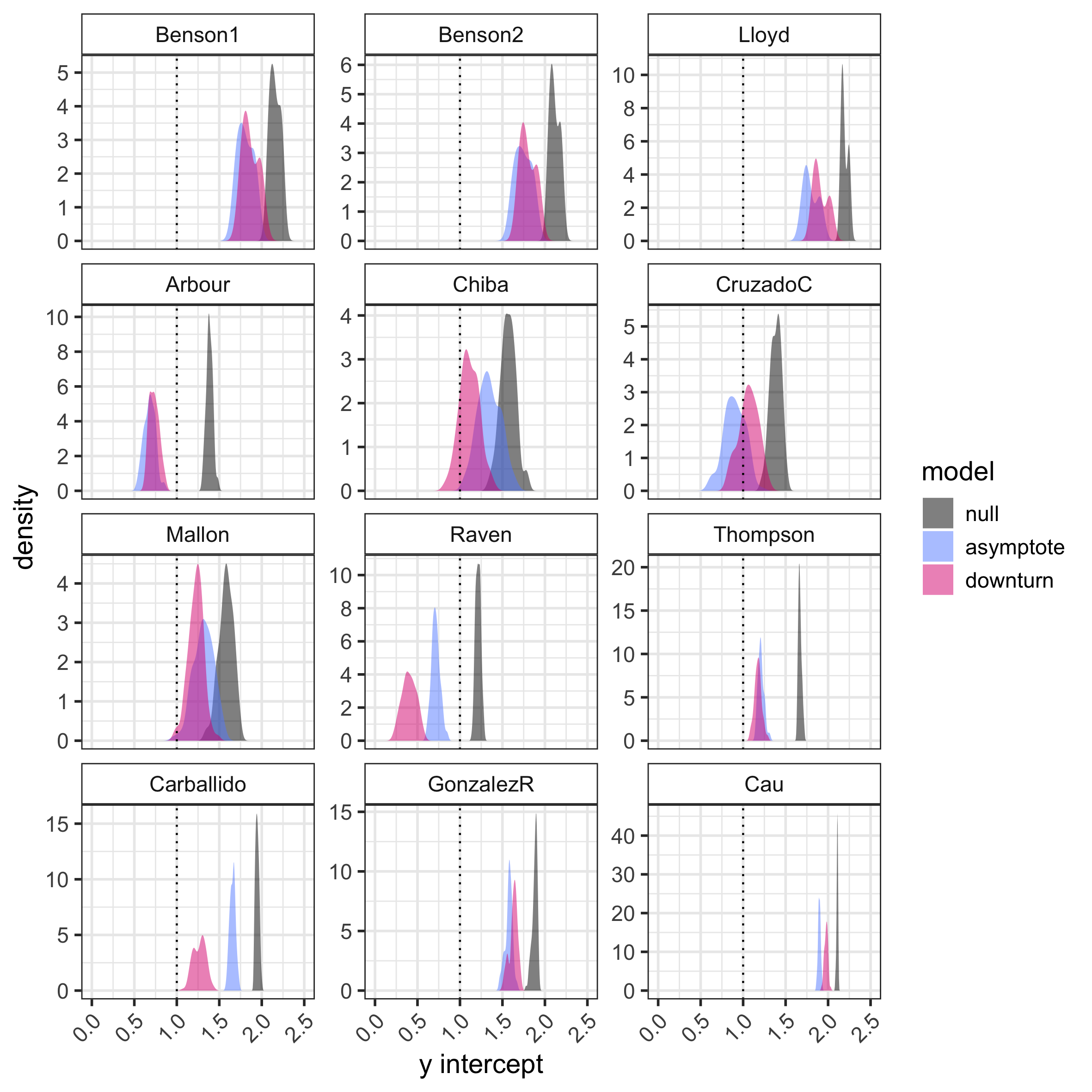
*

*Figure S5: Posterior mean y intercepts for the three models (see Fig 1) for each of 900 trees in this study, plus nine trees from Sakamoto et al. (2016), with intercepts estimated. Vertical lines show y intercepts equal to 1.0. The y-axis differs in each plot. Panels are ordered based on the dinosaur group in each tree as follows Dinosauria: Benson et al. (2014: two trees) and Lloyd et al. (2008); Ornithischia: Arbour et al. (2016), Chiba et al. (2018), Cruzado-Caballero et al. (2017), Mallon et al. (2016); Raven & Maidment (2017), Thompson et al. (2012); Sauropodomorpha: Carballido et al. (2017), Gonzàlez Riga et al. (2018); Theropoda: Cau et al. (2015).*

*
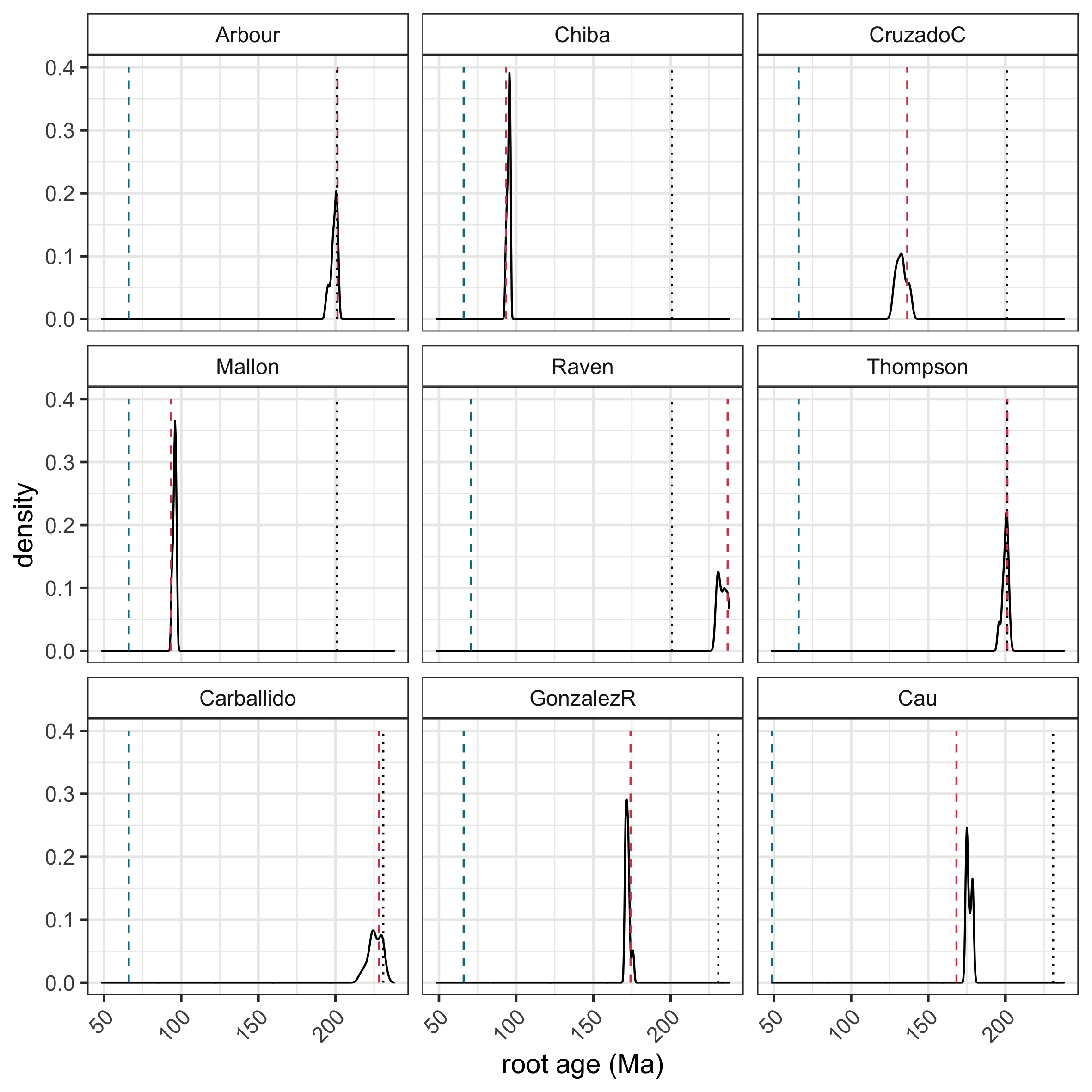
*

*Figure S6: Root ages for each of 900 trees in this study. The dotted black line shows the oldest inferred origin date for the clade, i.e. 201.3 Ma (Early Jurassic) for Ornithischia and 231.4 Ma (Carnian) for Sauropodomorpha and Theropoda. The red and blue dashed lines show the oldest and youngest ages respectively of taxa in the trees. Panels are ordered based on the dinosaur group in each tree as follows. Ornithischia: Arbour et al. (2016), Chiba et al. (2018), Cruzado-Caballero et al. (2017), Mallon et al. (2016); Raven & Maidment (2017), Thompson et al. (2012); Sauropodomorpha: Carballido et al. (2017), Gonzàlez Riga et al. (2018); Theropoda: Cau et al. (2015).*

**REFERENCES**

Arbour, V., Zanno, L. & Gates, T.A. 2016. Ankylosaurian dinosaur palaeo-environmental associations were influenced by extirpation, sea-level fluctuation, and geodispersal. *Palaeogeography Palaeoclimatology Palaeoecology*, **449**, 289-299.

Bapst, D.W., 2012. paleotree: an R package for paleontological and phylogenetic analyses of evolution. *Methods in Ecology and Evolution*, **3**, 803–807.

Benson RBJ, Campione NE, Carrano MT, Mannion PD, Sullivan C, Upchurch P, Evans DC. 2014 Rates of dinosaur body mass evolution indicate 170 million years of sustained ecological innovation on the avian stem lineage. *PLoS Biol.* **12**, e1001853.

Carballido, J.L., Pol, D., Otero, A., Cerda, I.A., Salgado, L., Garrido, A.C., Ramezani, J., Cúneo, N.R. & Krause, J.M. 2017. A new giant titanosaur sheds light on body mass evolution among sauropod dinosaurs. *Proceedings of the Royal Society B: Biological Sciences*, **284**, 20171219.

Cau, A., Brougham, T. & Naish, D. 2015. The phylogenetic affinities of the bizarre Late Cretaceous Romanian theropod Balaur bondoc (Dinosauria, Maniraptora): dromaeosaurid or flightless bird? *PeerJ*, **3**, 1032–1036.

Chiba, K., Ryan, M.J., Fanti, F., Loewen, M.A. & Evans, D.C. 2018. New material and systematic re-evaluation of *Medusaceratops lokii* (Dinosauria, Ceratopsidae) from the Judith River Formation (Campanian, Montana). *Journal of Paleontology*, **92**, 272–288.

Cruzado-Caballero, P. & Powell, J. 2017. Bonapartesaurus rionegrensis, a new hadrosaurine dinosaur from South America: implications for phylogenetic and biogeographic relations with North America. *Journal of Vertebrate Paleontology*, **37**, e1289381–17.

Goloboff, P. Farris, J. & Nixon, K. (2008). TNT, a free program for phylogenetic analysis. Cladistics, **24**. 774 - 786.

Gonzàlez Riga, B.J., Mannion, P.D., Poropat, S.F., Ortiz David, L.D. & Pedro Coria, J. 2018. Osteology of the Late Cretaceous Argentinean sauropod dinosaur *Mendozasaurus neguyelap*: implications for basal titanosaur relationships. *Zoological Journal of the Linnean Society,* **184**, 136-181.

Laurin, M. 2004. The evolution of body size, Cope's Rule and the origin of amniotes. *Systematic Biology,* **53**: 594-622.

Lloyd, G.T. Davis, K.E., Pisani, D., Tarver, J.E., Ruta, M., Sakamoto, M., Hone, D.W.E, Jennings, R., & Benton, M.J. 2008. Dinosaurs and the Cretaceous Terrestrial Revolution. *Proceedings of the Royal Society B: Biological Sciences*, **275**: 2483–2490.

Mallon, J.C., Ott, C.J., Larson, P.L., Iuliano, E.M. & Evans, D.C. 2016. *Spicylpeus shipporum*, gen. et sp. Nov., a boldly audacious new Chasmosaurine ceratopsid (Dinosauria: Ornithischia) from the Judith River formation (Upper Cretaceous: Campanian) of Montana, USA. *PLoS ONE* **11**(5): e0154218

R Core Team. 2019. R: A language and environment for statistical computing. R

Foundation for Statistical Computing, Vienna, Austria. https://www.R-project.org/.

Raven, T.J. & Maidment, S.C.R. 2017. A new phylogeny of Stegosauria (Dinosauria, Ornithischia). *Palaeontology*, **60:** 401-408.

Sakamoto, M., Benton, M.J. & Venditti, C. 2016. Dinosaurs in decline tens of millions of years before their final extinction. *Proceedings of the National Academy of Sciences USA*, **113**, 5036–5040.

Thompson, R.S., Parish, J.C., Maidment, S.C.R. & Barrett, P.M. 2012. Phylogeny of the ankylosaurian dinosaurs (Ornithischia: Thyreophora). *Journal of Systematic Palaeontology*, **10,** 301-312.
